# Supplementary material for: An integrated bioinformatic investigation of kallikrein gene family members in kidney renel cell carcinoma
Source: PLoS One. 2024 Aug 8;19(8):e0305070. doi: 10.1371/journal.pone.0305070 (PMC11309392; doi:10.1371/journal.pone.0305070)
Supplement: S1 Table — (DOCX) [file pone.0305070.s001.docx]

| Supplement Table 1.Results of GO KEGG enrichment analysis | | | | | | | |
| --- | --- | --- | --- | --- | --- | --- | --- |
| ONTOLOGY | ID | Description | GeneRatio | BgRatio | pvalue | p.adjust | qvalue |
| BP | GO:0016485 | protein processing | 10/34 | 328/18670 | 2.20e-10 | 3.18e-07 | 2.02e-07 |
| BP | GO:0051604 | protein maturation | 10/34 | 397/18670 | 1.40e-09 | 1.01e-06 | 6.42e-07 |
| BP | GO:0002576 | platelet degranulation | 7/34 | 128/18670 | 2.78e-09 | 1.34e-06 | 8.49e-07 |
| BP | GO:0022617 | extracellular matrix disassembly | 6/34 | 80/18670 | 6.25e-09 | 2.26e-06 | 1.43e-06 |
| BP | GO:0007597 | blood coagulation, intrinsic pathway | 4/34 | 18/18670 | 2.75e-08 | 7.95e-06 | 5.04e-06 |
| CC | GO:0031091 | platelet alpha granule | 7/34 | 91/19717 | 1.71e-10 | 2.13e-08 | 1.59e-08 |
| CC | GO:0031093 | platelet alpha granule lumen | 6/34 | 67/19717 | 1.53e-09 | 9.47e-08 | 7.08e-08 |
| CC | GO:0034774 | secretory granule lumen | 8/34 | 321/19717 | 5.68e-08 | 2.14e-06 | 1.60e-06 |
| CC | GO:0060205 | cytoplasmic vesicle lumen | 8/34 | 338/19717 | 8.45e-08 | 2.14e-06 | 1.60e-06 |
| CC | GO:0031983 | vesicle lumen | 8/34 | 339/19717 | 8.65e-08 | 2.14e-06 | 1.60e-06 |
| MF | GO:0004252 | serine-type endopeptidase activity | 17/34 | 160/17697 | 1.54e-26 | 1.88e-24 | 1.23e-24 |
| MF | GO:0008236 | serine-type peptidase activity | 17/34 | 182/17697 | 1.51e-25 | 8.99e-24 | 5.89e-24 |
| MF | GO:0017171 | serine hydrolase activity | 17/34 | 186/17697 | 2.21e-25 | 8.99e-24 | 5.89e-24 |
| MF | GO:0004175 | endopeptidase activity | 17/34 | 427/17697 | 3.72e-19 | 1.14e-17 | 7.44e-18 |
| MF | GO:0004866 | endopeptidase inhibitor activity | 6/34 | 175/17697 | 9.17e-07 | 1.76e-05 | 1.15e-05 |
| KEGG | hsa04610 | Complement and coagulation cascades | 7/18 | 85/8076 | 3.23e-10 | 3.13e-08 | 2.51e-08 |
| KEGG | hsa05215 | Prostate cancer | 5/18 | 97/8076 | 1.71e-06 | 8.27e-05 | 6.64e-05 |
| KEGG | hsa05205 | Proteoglycans in cancer | 4/18 | 205/8076 | 9.33e-04 | 0.025 | 0.020 |
| KEGG | hsa04614 | Renin-angiotensin system | 2/18 | 23/8076 | 0.001 | 0.025 | 0.020 |
| KEGG | hsa04750 | Inflammatory mediator regulation of TRP channels | 3/18 | 100/8076 | 0.001 | 0.025 | 0.020 |
| KEGG | hsa04919 | Thyroid hormone signaling pathway | 3/18 | 121/8076 | 0.002 | 0.037 | 0.029 |
| KEGG | hsa04915 | Estrogen signaling pathway | 3/18 | 138/8076 | 0.003 | 0.041 | 0.033 |
| KEGG | hsa05418 | Fluid shear stress and atherosclerosis | 3/18 | 139/8076 | 0.003 | 0.041 | 0.033 |
| KEGG | hsa05224 | Breast cancer | 3/18 | 147/8076 | 0.004 | 0.043 | 0.034 |
| KEGG | hsa04961 | Endocrine and other factor-regulated calcium reabsorption | 2/18 | 53/8076 | 0.006 | 0.059 | 0.047 |
| KEGG | hsa04510 | Focal adhesion | 3/18 | 201/8076 | 0.009 | 0.075 | 0.060 |
| KEGG | hsa04015 | Rap1 signaling pathway | 3/18 | 210/8076 | 0.011 | 0.075 | 0.060 |
| KEGG | hsa04520 | Adherens junction | 2/18 | 71/8076 | 0.011 | 0.075 | 0.060 |
| KEGG | hsa04115 | p53 signaling pathway | 2/18 | 73/8076 | 0.011 | 0.075 | 0.060 |
| KEGG | hsa05133 | Pertussis | 2/18 | 76/8076 | 0.012 | 0.075 | 0.060 |
| KEGG | hsa05100 | Bacterial invasion of epithelial cells | 2/18 | 77/8076 | 0.012 | 0.075 | 0.060 |
| KEGG | hsa01521 | EGFR tyrosine kinase inhibitor resistance | 2/18 | 79/8076 | 0.013 | 0.075 | 0.060 |
| KEGG | hsa04919 | Thyroid hormone signaling pathway | 3/18 | 121/8076 | 0.002 | 0.037 | 0.029 |
