# Supplementary material for: An integrated bioinformatic investigation of kallikrein gene family members in kidney renel cell carcinoma
Source: PLoS One. 2024 Aug 8;19(8):e0305070. doi: 10.1371/journal.pone.0305070 (PMC11309392; doi:10.1371/journal.pone.0305070)
Supplement: Attachment — Submitted filename: Response to Reviewers.docx [file pone.0305070.s006.docx]

Guang’anmen Hospital, China Academy of Chinese Medical Sciences

Beijing, China, 5/03/2024

Dear Editors and Reviewers:

Thank you for your letter about our manuscript entitled ‘An Integrated Bioinformatic Investigation of Kallikrein Gene Family Members in Kidney Renel Cell Carcinoma’(ID:PONE-D-23-25080). Those comments are very helpful for improving our paper. We have improved the paper along the lines suggested by those comments which we hope meet wtih approval. The revised portion about the manuscript are marked in blue in this letter. The main corrections in the paper and the point-by-point response to comments are as follows:

Journal Requirements:

1. *Please review your reference list to ensure that it is complete and correct. If you have cited papers that have been retracted, please include the rationale for doing so in the manuscript text, or remove these references and replace them with relevant current references. Any changes to the reference list should be mentioned in the rebuttal letter that accompanies your revised manuscript. If you need to cite a retracted article, indicate the article’s retracted status in the References list and also include a citation and full reference for the retraction notice.*

Response: Thank you for your reply. We have revised the manuscript as follow.

"44.Jing X, Qin X, Liu H, Liu H, Wang H, Qin J, et al. DNA damage response alterations in clear cell renal cell carcinoma: clinical, molecular, and prognostic implications. Eur J Med Res. 2024 Feb 7;29(1):107. doi: 10.1186/s40001-024-01678-x."

Editor Comments:

1. *I strongly suggest that the authors need to deposit their codes for R packages DESeq2, edgeR and clusterProfiler etc in github or appendix.*

Response: Thank you for your suggestion. We have supplemented the relevant codes for R packages in zip file named "demo.R".

Many thanks for your time and consideration.

Yours Sincerely,

Baoquan Wang

Guang’anmen Hospital, China Academy of Chinese Medical Sciences

Tel: +86 18810216061	Email: 20200931825@bucm.edu.cn
